# Autophagy Dysregulation in Crohn’s Disease and Colorectal Cancer—An Analysis of *BECN1*, *PINK1*, and *LAMP2* Gene Expression

**DOI:** 10.3390/cimb48010031

**Published:** 2025-12-26

**Authors:** Magda Bichalska-Lach, Dariusz Waniczek, Paweł Kowalczyk, Mirosław Śnietura, Mariusz Kryj, Martyna Bednarczyk, Małgorzata Muc-Wierzgoń

**Affiliations:** 1 Department of Surgical Nursing and Propaedeutics of Surgery, Faculty of Health Sciences in Katowice, Medical University of Silesia, 40-055 Katowice, Poland; mbichalska@sum.edu.pl; 2 Department of Oncological Surgery, Faculty of Medical Sciences in Zabrze, Medical University of Silesia, 40-055 Katowice, Poland; mariusz.kryj@sum.edu.pl; 3 Department of Animal Nutrition, The Kielanowski Institute of Animal Physiology and Nutrition, Polish Academy of Sciences, Instytucka 3, 05-110 Jabłonna, Poland; 4 Chair and Department of Pathomorphology and Molecular Diagnostics, Faculty of Medical Sciences in Katowice, Medical University of Silesia, 40-055 Katowice, Poland; 5 Department of Hematology and Cancer Prevention, Medical University of Silesia, 40-055 Katowice, Poland; martyna.bednarczyk@sum.edu.pl; 6 Department of Internal Diseases, Propedeutics and Emergency Medicine, Faculty of Public Health in Bytom, Medical University of Silesia in Katowice, Piekarska 18, 44-902 Bytom, Poland; mwierzgon@sum.edu.pl

**Keywords:** autophagy, *BECN1*, Crohn’s disease, colorectal cancer, *LAMP2*, *PINK1*

## Abstract

Crohn’s disease (CD) and colorectal cancer (CRC) are clinically distinct but pathogenetically related conditions in which significant abnormalities in autophagy are observed. The aim of the study was to evaluate the expression of three key autophagy-related genes, i.e., *BECN1* (macroautophagy), *PINK1* (mitophagy), and *LAMP2* (chaperone-mediated autophagy) in tissue samples from patients with CD and CRC. The study material included samples from 48 patients with CD (*n* = 96 biopsy samples) and 87 patients with CRC (*n* = 87 tumors; *n* = 87 normal paired controls). Transcriptomic analyses were performed using Affymetrix HG-U133A microarrays. They were confirmed by RT-qPCR. The Kruskal–Wallis test with Dunn’s post hoc analysis (α = 0.05) and Spearman’s correlation coefficients were used for statistical evaluation. Expression of *BECN1* and *LAMP2* was significantly decreased in both CD and CRC compared to the controls (*p* = 0.009; *p* = 0.023, respectively). However, *PINK1* showed significantly higher expression levels in CD compared to CRC and the controls (*p* < 0.001). The clinical stages of CRC (I–IV) did not significantly affect the expression of the analyzed genes. The study findings confirm the presence of common abnormalities in autophagy in CD and CRC, with decreased macroautophagy and chaperone-mediated autophagy, with the compensatory activation of mitophagy. *BECN1*, *PINK1*, and *LAMP2* expressions may have a diagnostic and therapeutic value in the context of chronic inflammation and colorectal carcinogenesis.

## 1. Introduction

Crohn’s disease (CD) is a chronic inflammatory bowel disease (IBD) associated with an increased risk of colorectal cancer (CRC). Long-standing intestinal inflammation, epithelial damage, and impaired immune regulation contribute to inflammation-driven carcinogenesis. Epidemiological studies indicate that patients with IBD have approximately a two-fold higher risk of CRC compared with the general population, with cumulative risk increasing with disease duration, extent of colonic involvement, and coexisting conditions such as primary sclerosing cholangitis. CRC remains one of the leading causes of mortality in IBD patients, underscoring the importance of identifying molecular mechanisms linking chronic intestinal inflammation and tumorigenesis [1,2,3,4,5,6,7,8,9,10,11,12].

Autophagy is one of the primary mechanisms of cellular homeostasis and occurs by recycling damaged proteins and organelles and older or misfolded proteins [13,14,15]. The levels of autophagy can be impaired depending on various factors, such as inflammatory diseases and cancer [16,17]. It seems that autophagy can be both “an enemy and ally” of cancer, depending on pathological or physiological conditions [18]. This appears to be associated with autophagy (macroautophagy, microautophagy, selective autophagy, chaperone-mediated autophagy) [19]. Macroautophagy is a process consisting of several stages in which the BECN1 protein is responsible for the formation of the phagophore, which leads to the formation of the autophagosome.

In turn, the *PINK1* protein is involved in lysosomal mitochondrial degradation in selective autophagy (mitophagy), and the *LAMP2* protein is involved in chaperone-mediated autophagy (CMA) regulation [20,21]. Autophagy processes are impaired in CD and CRC. Inhibition of autophagy is predominant in CD, which results in the overproduction of pro-inflammatory cytokines that exacerbate chronic inflammation, while in CRC, autophagy is changed from a protective function to a pro-tumor activity, which promotes tumor growth and resistance to treatment. Understanding these mechanisms opens new therapeutic possibilities [13].

The aim of the study was to investigate the potential relationship between autophagy dysregulation in patients with CD and those with colorectal cancer. Specifically, we compared the expression of genes encoding key autophagy-related proteins (*BECN1*, *PINK1*, and *LAMP2*) to identify similarities or differences in transcriptional activity between the two patient groups. The analysis of gene expression levels may provide insights into the shared molecular mechanisms underlying impaired autophagy in chronic intestinal inflammation and tumorigenesis. Our findings may contribute to a better understanding of the dual role of autophagy in IBD and colorectal cancer development.

This study builds upon our previous investigations into transcriptional alterations of autophagy-related genes in colorectal cancer, extending the analysis to inflammatory bowel disease to explore shared molecular mechanisms linking chronic inflammation and carcinogenesis [22,23,24].

Given the complexity of autophagy regulation, this study focused on three key genes representing distinct but complementary autophagy pathways: *BECN1*, a central regulator of macroautophagy initiation; *PINK1*, a key mediator of selective mitochondrial autophagy (mitophagy); and *LAMP2*, an essential component of chaperone-mediated autophagy (CMA). These genes were selected based on prior transcriptomic analyses and experimental evidence indicating that they exhibit consistent and potentially relevant expression changes in colorectal cancer and inflammatory conditions.

By targeting genes that reflect mechanistically distinct autophagy processes, we aimed to capture a broader picture of autophagy dysregulation linking chronic intestinal inflammation and colorectal carcinogenesis.

Although gene expression analysis does not directly measure autophagic flux, transcriptional dysregulation of key autophagy-related genes may reflect altered cellular capacity to initiate or regulate specific autophagy pathways. In the tissue context of chronic inflammation or cancer, sustained changes in mRNA levels may therefore indicate long-term impairment or adaptive remodeling of autophagy mechanisms.

## 2. Materials and Methods

### 2.1. Patients and Methods

#### 2.1.1. Study Design

The study included 135 patients (aged 38 to 83 years), 87 of whom underwent elective surgery for CRC at different clinical stages of the disease, and 48 patients diagnosed with CD. In patients with CD, the Crohn’s Disease Activity Index (CDAI) was determined (median 188, range 74–446). The clinical characteristics of the study group with CD and CRC are given in Table 1, Table 2 and Table 3.

The inclusion criteria for patients with CD and CRC were as follows: age > 18 years, any stage of disease progression and written informed consent to participate in the study.

The exclusion criteria were as follows: severe systemic or metabolic conditions (except for obesity as an isolated disorder), a history of radio- or chemotherapy, other malignant conditions, active or a history of chronic inflammatory conditions, including IBD in patients with CRC and second surgery for the underlying disease.

Tumor and healthy control tissue samples were obtained during classical surgical resection of the colon due to cancer. The material consisted of tumor and healthy tissue samples (control samples *n* = 87; control tissues for CD were taken from macroscopically unaffected areas of the intestine, distant from the inflamed lesions, to serve as internal controls.) obtained from an area at least 5 cm outside the histologically negative margin. Cancer samples were obtained from the peripheral regions of the tumor to exclude the presence of necrotic tissue.

All samples were obtained by the same surgical team to minimize errors. In patients with CD, two samples of the affected tissue were taken from each patient during a colonoscopy (*n* = 43 sample pairs). Two samples were taken from the visible lesion in each patient with CD (*n* = 5) undergoing elective surgery for (sub)ileus or internal fistula (*n* = 5 sample pairs). In patients with CD, two samples of affected tissue were collected due to the difficulties in precisely determining the location of the most severe intestinal inflammatory lesions during the endoscopic examination and blurring of the macroscopic boundaries between healthy and affected tissue, which resulted in obtaining twice as many results as the real number of patients in the group. Immediately after the excision of the colonic segment or endoscopic biopsy collection, the material was placed in sterile tubes containing RNA later^TM^ (Sigma, Toronto, ON, Canada) in the amount of 10 µL per 1 mg of tissue (200 µL RNA later^TM^ per 20 mg of tissue). The samples were stored for 24 h at 4 °C. Next, the sections were frozen at −80 °C until further analysis. Molecular studies were performed at the Department of Molecular Biology of the Medical University of Silesia.

#### 2.1.2. Bioethical Consent

The study received the consent of the Medical University of Silesia (KB SUM No. KNW/0022/KB1/21/I/10). All authors are committed to complying with the ethical principles of clinical research based on the Declaration of Helsinki.

### 2.2. Methods

Eighty-seven paired tissues (87 tumor and 87 control samples) were collected from patients with CRC. A total of 96 pathologically changed tissues were obtained from patients with CD.

#### 2.2.1. RNA Isolation and RT-qPCR

The first step was to isolate the total RNA. The tissue material was homogenized using an electric homogenizer (Kinematica AG, Bern, Switzerland). The RNA was isolated according to the manufacturer’s instructions using the TRIzol^®^ reagent (Life Technologies, Carlsbad, CA, USA). RNA was purified with the Qiagen RNeasy Mini Kit (Qiagen, Hilden, Germany) in combination with DNase I digestion. RNA integrity was assessed using the spectrophotometric and electrophoretic quality control methods. RIN (RNA Integrity Number) values were measured for all samples, and only samples with RIN ≥ 7 were included in the analyses. The Gene Quant II (Pharmacia Biotech, Uppsala, Sweden) spectrophotometer was used to quantify the RNA using an absorbance of 260 nm. Confirmation of the results of the comparative analysis of transcriptomes determined by the expression microarray technique was carried out using the RT-qPCR method, which is considered the gold standard in the validation of matrix experiments. The results of the transcriptional activity analysis are given as the number of mRNA copies per 1 μg of total RNA.

The thermal profile of the RT-qPCR reaction included the following steps: reverse transcription (45 °C for 10 min), polymerase activation (95 °C for 2 min), 40 cycles including denaturation (95 °C for 5 s), primer attachment (60 °C for 10 s), and elongation (72 °C for 5 s). The reaction was performed using sequence-specific primer pairs for each gene (Sigma-Aldrich, St. Louis, MO, USA). The primer sequences for BECN1, LAMP2, and PINK1 are listed in Table 4.

The expression of genes involved in ubiquitin-mediated protein degradation was investigated. The quantitative mRNA amplification reaction was performed for the following genes: *BECN1*, *PINK1*, and *LAMP2*. The number of mRNA molecules of the investigated genes was calculated based on the standard curve prepared for commercially available DNA templates of the β-actin gene using the TaqMan DNA Template Reagent (PE Applied Biosystems, Foster City, CA, USA—850 Lincoln Centre Drive, Foster City, CA 94404, USA) in five concentrations (400, 800, 2000, 4000, and 8000 copies of β-actin cDNA/μL). On the basis of the fluorescence curve recorded after each amplification cycle, the number of mRNA copies for each target gene was calculated per 1 μg of RNA.

#### 2.2.2. Microarray Analysis

Microarray analysis was validated with qRT-PCR. The transcriptional activity of genes involved in autophagy in CD and CRC was investigated and compared to the normal tissues (controls). Transcriptomic analysis was performed using Affymetrix HG-U133A oligonucleotide microarrays. Tumor and matched control tissue samples from patients with colorectal cancer, as well as intestinal tissue samples from patients with Crohn’s disease, were processed according to the manufacturer’s instructions.

Raw microarray intensity data were background-corrected and normalized using the Robust Multi-array Average (RMA) algorithm. Normalized expression values were obtained using GeneSpring GX 11.5 software (Agilent Technologies, Santa Clara, CA, USA—5301 Stevens Creek Boulevard, Santa Clara, CA 95051, USA).

The microarray analysis was used to assess transcriptional activity of selected autophagy-related genes and to guide subsequent RT-qPCR validation. RT-qPCR constituted the primary quantitative method used for statistical comparisons in the present study.

### 2.3. Limitations of the Study

This study has several limitations. Firstly, expression analysis was limited to the mRNA level (qPCR) without simultaneous assessment of functional autophagy activity, such as LC3 or p62 protein levels and autophagosome flux dynamics. Secondly, the lack of longitudinal data makes it impossible to assess changes in autophagy gene expression over time, particularly during the transition from CD to CD-CRC, which limits the ability to identify these genes as early markers of tumor transformation.

Due to limited sample sizes within CRC stages, statistical power for stage-based comparisons was limited, particularly for stage IV.

In addition, translation and protein expression levels were not assessed, which is a significant limitation since a decrease in mRNA levels is not necessarily directly associated with a decrease in functional protein levels and that future studies including protein-level analyses (e.g., Western blot, immunohistochemistry) are needed to confirm the functional relevance of *BECN1*, *PINK1*, and *LAMP2* in CD and CRC.

Furthermore, the study groups differed in terms of age, weight, and BMI due to their different clinical nature. However, these variables may influence gene expression patterns and should be considered when interpreting the results.

Trends observed across cancer stages should be interpreted with caution.

Finally, although preliminary bioinformatic analyses of publicly available datasets (e.g., GEO) rather than extensive analyses could provide additional validation of our findings, the present study was designed as a research objective- and hypothesis-driven continuation of our previous work, focusing specifically on three genes: *BECN1*, *PINK1*, and *LAMP2*. Therefore, we prioritized targeted validation of these genes over genome-wide screening. Integration of our results with publicly available datasets remains an important avenue for ongoing research.

### 2.4. Statistical Analysis

#### 2.4.1. Experimental Statistical Analysis

Statistical analysis of RT-qPCR results was performed using IBM SPSS Statistics version 26.0 (IBM Corp., Armonk, NY, USA). Gene expression levels were analyzed to assess differences in the transcriptional activity of autophagy-related genes between study groups. For each parameter, descriptive statistics were calculated, including mean, median, minimum and maximum values, standard deviation, and lower (25%) and upper (75%) quartiles. Group comparisons were conducted using the Kruskal–Wallis H test. When statistically significant differences were detected, post hoc comparisons were performed using Dunn’s test with Bonferroni correction. A *p*-value < 0.05 was considered statistically significant.

#### 2.4.2. Supportive Bioinformatic Analysis

In addition to the primary statistical analysis of experimentally generated data, supportive bioinformatic analyses were conducted to provide biological context for the investigated autophagy-related genes.

Autophagy-related genes were initially identified using the Human Autophagy Database (HADb). Publicly available gene expression datasets relevant to inflammatory bowel disease and colorectal cancer (including *GSE*43292 and *GSE*28829) were consulted to review previously reported expression patterns of autophagy-associated genes and were not independently reprocessed.

Selected datasets were explored using established bioinformatic approaches as described in the original dataset publications. Functional annotation and pathway context were reviewed using Gene Ontology (GO) terms and Kyoto Encyclopedia of Genes and Genomes (KEGG) pathway annotations, with particular emphasis on biological processes related to autophagy, lysosomal function, mitochondrial quality control, and regulation of cell death.

Protein–protein interaction networks involving autophagy-related proteins were reviewed using the STRING database to illustrate known and predicted molecular interactions among key regulators of autophagy, including *BECN1*, *PINK1*, and *LAMP2*, and their associated signaling partners.

These supportive bioinformatic analyses were not used for independent hypothesis testing, statistical validation, or result generation, but served exclusively as background information to aid biological interpretation and contextualization of the experimental findings obtained in the present study.

## 3. Results and Discussion

In the first step, the basic descriptive statistics for the analyzed gene expression were compared in three groups: CD, CRC, and control tissue samples. Table 5 shows the descriptive statistics for *BECN1*, *PINK1* and *LAMP2* in the analyzed groups.

The medians for *BECN1* and *LAMP2* in the CD and CRC groups were lower than in the control group. On the other hand, *PINK1* had a very high median expression in patients with CD. A significantly lower median expression of *PINK1* was reported in the group of patients with CRC, which was also higher than in the control group.

**Figure 1:**
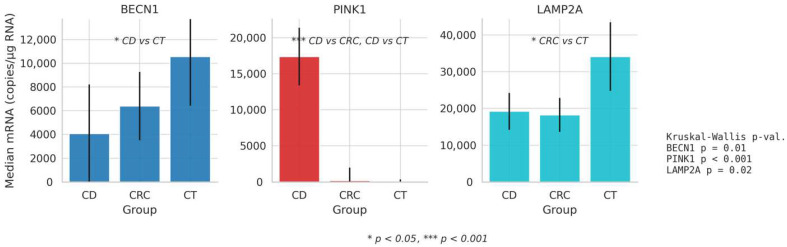
Graphical version of comparison of gene *BECN1*, *PINK1*, *LAMP2* mRNA expression in CD, CRC, and control groups using the Kruskal–Wallis H test.

The Kruskal–Wallis H test was performed to compare the results of patients with CD and CRC with the controls. The analysis showed significant differences in the values of *BECN1*, *PINK1*, and *LAMP2* between the groups. To determine the differences between the compared groups, Dunn’s post hoc pairwise comparisons test with a Bonferroni correction was applied in Table 6 and Figure 1.

Significantly higher levels of *BECN1* were found compared to the control group (*p* = 0.009). Samples of patients with CD showed higher levels of *PINK1* compared to those with CRC (*p* <0.001) and the controls (*p* < 0.001). Significantly lower levels of *LAMP2* were found in CRC samples compared to the controls (*p* = 0.023). Other differences between the groups were non-significant.

**Figure 2:**
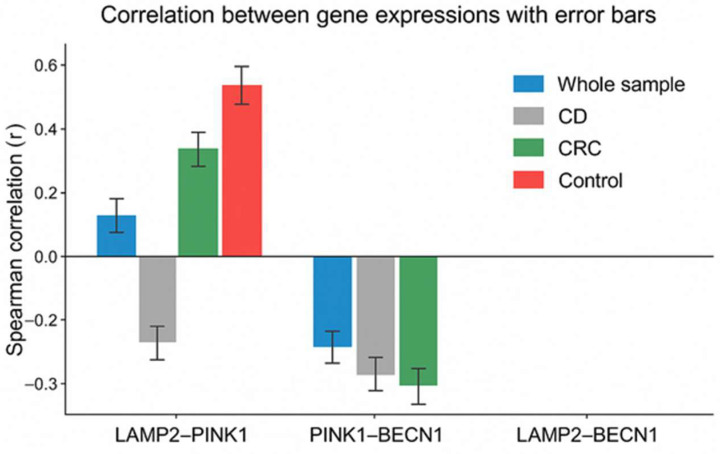
Expressions of the three genes using Spearman correlation analysis.

In addition, correlation analysis was performed between the expressions of the three genes using Spearman correlation analysis (Table 7 and Figure 2). Correlation coefficients between *LAMP2*, *PINK1*, and *BECN1* expression levels were evaluated across the entire sample and within each subgroup (CD, CRC, and controls).

In the whole sample, a weak but statistically significant positive correlation was observed between *LAMP2* and *PINK1* (r = 0.16, *p* < 0.01), while *PINK1* and *BECN1* were negatively correlated (r = −0.23, *p* < 0.01). No significant correlation was found between *LAMP2* and *BECN1* in this group. No statistically significant correlations were detected between the analyzed genes in patients with CD. In the CRC group, *LAMP2* and *PINK1* showed a moderate positive correlation (r = 0.28, *p* < 0.01), whereas *PINK1* and *BECN1* were inversely correlated (r = −0.24, *p* < 0.05). The correlation between *LAMP2* and *BECN1* was not significant. Interestingly, a strong positive correlation was found between *LAMP2* and *PINK1* (r = 0.51, *p* < 0.01) in the control group, which was the most pronounced among all subgroups. No significant correlations were reported between *BECN1* and the other two genes in this group (Figure 2).

**Figure 3:**
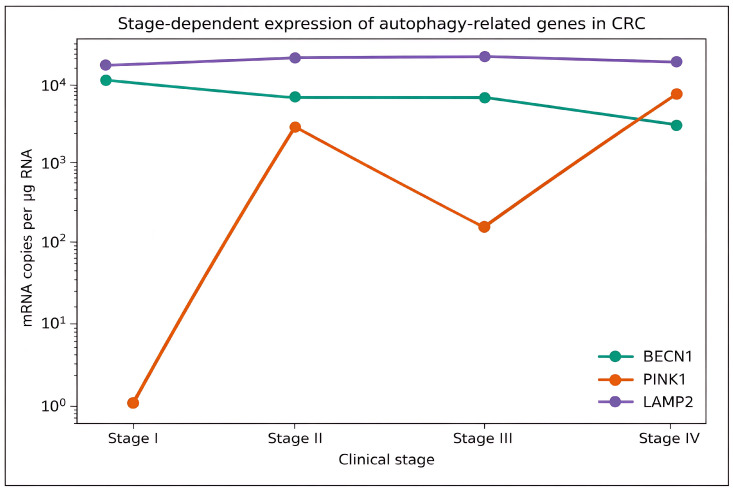
The Kruskal–Wallis H test was used to compare gene expression across colon cancer stages (CSI, CSII, CSIII, and CSIV). Legend: *BECN1*—decreasing from Stage I to IV (green square). *PINK1*—non-linear increase, highest in Stage IV (red square). *LAMP2*—increases to Stage III, slight decrease in Stage IV (plum square). Each stage has three bars grouped (one for each gene). Bars are labeled with median mRNA copies for more straightforward interpretation. Y-axis is mRNA copies/μg, X-axis is tumor stage (I–IV).

Subsequently, the Kruskal–Wallis H test was performed to compare gene expression among samples with different stages of colon cancer (CSI, CSII, CSIII and CSIV). The detailed results of the analysis are shown in Table 8 and Figure 3.

Although trends were observed, no statistically significant differences were found between stages for the analyzed genes. For *BECN1*, the median expression decreased progressively from stage I (10,245 mRNA copies/μg) to stage IV (2629 mRNA copies/μg), which suggests a potential downregulation in advanced disease. However, this trend did not reach statistical significance (*H* = 4.21, *p* = 0.240; *η*^2^ < 0.01).

In contrast, *PINK1* expression showed a non-linear increase, with the highest median found in stage IV (7203mRNA copies/μg) and the lowest in stage I (1.4mRNA copies/μg). The difference was not statistically significant (*H* = 4.70, *p* = 0.196; *η*^2^ = 0.01).

For *LAMP2*, a gradual increase in expression was noted from stage I (14,735 mRNA copies/μg) to stage III (19,830 mRNA copies/μg) to decrease in stage IV to 17,775 mRNA copies/μg. Again, the differences between the groups were not statistically significant (*H* = 1.86, *p* = 0.602; *η*^2^ < 0.01).

Overall, the analysis suggests that although slight trends in gene expression across tumor stages could exist, these differences were not statistically or biologically significant in the cohort (Figure 3).

Given the multifactorial pathogenesis of CRC and IBD, dysregulation of autophagy has been proposed as one of the mechanisms linking chronic inflammation with carcinogenesis. Mutations, genetic instability, epigenetic changes, impaired immune response by mucosal inflammatory mediators, oxidative stress, and intestinal microbiota are thought to be responsible for CRC and IBD [25,26,27].

Abnormal autophagy processes are also observed in both CRC and CD. However, their roles in the pathogenesis of these conditions are different. In both cases, autophagy disorders result from or lead to exacerbation of the disease. In CD, autophagy generally has a protective function since it maintains cellular homeostasis and limits excessive inflammatory response [28,29,30,31]. Ineffective autophagy results in uncontrolled inflammation, damage to the intestinal barrier, and disease progression, as confirmed by genetic studies indicating that polymorphisms in autophagy genes (e.g., *ATG16L1*, *IRGM*, *NOD2*) correlate with increased susceptibility to CD [29,31].

In turn, altered autophagy and chronic inflammation in CRC may promote neoplastic transformation by changing the inflammatory or immunosuppressive tumor microenvironment [27,32,33]. Accumulation of damaged organelles, proteins, and toxic metabolites in intestinal epithelial cells promotes induction of oxidative stress and DNA damage. Genomic instability is a key starting point for initiating and promoting tumor transformation by facilitating mutations in genes responsible for tumor growth inhibition and cell cycle control. Furthermore, in tumor-transformed cells, dysregulation of autophagy may enhance their ability to survive under unfavorable conditions (e.g., limited nutrient availability) and can affect resistance to anticancer treatment [21,26,27,34].

In this respect, the aim of normal autophagy is to protect the body from excessive DNA damage, which directly contributes to the inhibition of carcinogenesis. Therefore, it may play a crucial role in this process. For example, DNA damage to cells affected by chronic inflammation can inhibit one of the major cell proliferation pathways (mTOR). Its inhibition is a stimulus for the activation of autophagy processes aimed at degrading damaged cells, thereby preventing their potential malignant transformation [26,35].

Autophagy is responsible for destroying cells with damaged DNA and prevents processes that can lead to mutation or damage to DNA. The phenomenon of mitophagy is responsible for it, which is the process of removing damaged mitochondria that can generate reactive oxygen species (mutagenic agents) [36]. Disorders of autophagy contribute to the occurrence of mutations in genes encoding key proteins of autophagy, which exacerbate the disorders [37,38,39]. As in the case of chronic inflammatory diseases, the aging body shows reduced activity or inhibition of autophagy processes [40]. Furthermore, autophagy abnormalities in the elderly and in the course of chronic inflammatory diseases may contribute to an increased risk of developing cancers, including CRC and cancers coexisting with CD [41,42].

In addition, defects in autophagy genes associated with CD and CRC were found. Mutations in autophagy-related genes, such as *ATG16L1* and *IRGM*, play an essential role in the development of CD and may increase the risk of CRC. The resulting abnormalities in secretory cells (Paneth and cup cells) weaken the intestinal barrier and increase the inflammatory process. Additionally, *ATG16L1* and *IRGM* gene variants may also be associated with abnormal morphology and mitochondrial dysfunction [28,29,42,43].

Therefore, changes can lead to the dysfunction of crucial immune intestinal mechanisms and increased oxidative stress. Long-term inflammation associated with impaired autophagy can lead to neoplastic lesions under favorable conditions [43,44]. These defects in autophagy mean that initially “anticancer” processes can transform into ones that favor the promotion and proliferation of cancer cells. This is due to the accumulation of toxic proteins, increased oxidative stress, or disruption of host-microbiota interactions [25,26,28,42,43,44]. Many proteins are involved in regulating autophagy, of which Beclin-1 (*BECN1*), *PINK1* and *LAMP2* are crucial for different types of autophagy. Our study determined transcription levels in three genes encoding autophagy proteins: *BECN1*, *PINK1* and *LAMP2*, representing three of the four types of autophagy in the body.

Macroautophagy plays a major role in cell degradation. It prevents the accumulation of cytotoxic components [45]. *BECN1* encoding the Beclin-1 protein is a crucial gene in macroautophagy, which was identified in chromosome 17q21 [46]. *BECN-1* is a tumor suppressor gene and plays a role in tumorigenesis, neurodegeneration, apoptosis and autophagy [25,46,47,48]. Studies have suggested an association between *BECN1* expression and early-stage cancer, although this association is not direct [48].

It is believed that the limited expression of *BECN1* is positively correlated with a worse prognosis in some cancers, including gastric cancer [49]. According to other reports, *BECN-1* expression is inversely associated with metastases, venous infiltration, TNM staging, differentiation and a favorable prognosis of gastric cancer [18]. In addition, reduced expression of *BECN1* promotes breast, liver, lung and ovarian cancers. Mutations in the gene encoding the *BECN-1* protein are associated with the progression of these cancers [47,48,49,50,51]. It was also reported that *BECN1* levels could be elevated in ovarian, prostate, or breast cancers [46,47,48,51]. *BECN1* overexpression in ovarian cancer was negatively correlated with differentiation and higher cumulative recurrence-free survival rates [51].

BECN-1 protein is mainly described as anti-oncogenic. It also prevents inflammatory processes in the intestine. Increased autophagy induced by *BECN1* had complex protective and anti-inflammatory effects in the intestine in experimental models and clinical observations of CD and IBD [28,52,53].

A similar observation was found in our study. *BENC1* expression was lower in CD and CRC than in healthy tissue. Although our study showed lower expression of this gene in tumor tissue, the expression was not different in the early stages from the expression in unaffected tissue. Decreasing expression levels in further disease stages would indicate the greater importance of this process in tumor progression at a later stage. This suggests that the further “breakdown of macroautophagy” determined by *BENC1* may strongly influence its higher expression, while maintaining normal BECN-1-dependent autophagy promotes the integrity of the intestinal barrier, limits excessive activation of the immune system and inflammatory processes, and protects cells from cell damage and tumor progression.

This also confirms a marked decrease in *BECN1* activity observed in CD, with a lower mean gene copy number in affected tissues (4062.0) compared to CRC samples (6388.0). This may indicate a different dynamic of potential carcinogenesis in CD, possibly favoring early and rapid tumor dissemination [54]. It may explain a worse prognosis of patients with CRC based on CD or IBD, especially with a long-term disease. A long-term course of CD increased the risk of CRC, and cancer developing on this basis is usually diagnosed at more advanced stages. There are also data indicating that IBD-CRC is more biologically aggressive, more locally advanced, and has a lower percentage of R0 resection, which translates into a worse prognosis and higher mortality [10,55,56]. Other reports also showed that the low expression of *BECN1* was associated with liver metastasis and distant CRC metastases [57].

Selective autophagy, known as mitophagy, removes damaged mitochondria from cells [28]. Mitochondria are an essential source of ATP for cell function [39]. Through autophagic quality control mechanisms, a significant percentage of damaged or old mitochondria are replaced daily, which allows cells to maintain healthy mitochondria, preventing excessive inflammation and oxidative stress and being beneficial for cellular homeostasis [58,59].

Mitophagy is activated by hypoxia, nutrient deficiency, DNA damage, inflammation and mitochondrial membrane depolarization. The *PINK1* gene encodes the serine/threonine protein kinase PINK1, which localizes to mitochondria and is thought to protect cells from stress-induced mitochondrial dysfunction [60,61,62]. *PINK1* directly contributes to mitochondrial breakdown by several mechanisms [63,64]. Studies have shown deletion of the gene encoding *PINK1* in CRC, esophageal cancer, brain cancer, breast cancer, head and neck cancer, liver carcinoma, ovarian cancer, melanoma, and leukemia, which can induce carcinogenesis due to mitochondrial dysfunction [47,62,65]. However, *PINK1* mRNA expression was significantly increased in lymphoma [62].

In our study, *PINK1* expression had a very high median expression in the CD group with significantly lower median levels of *PINK1* expression in CRC patients and controls (*p* < 0.001). The median of patients with CRC was higher than in the controls, although the difference was not statistically significant. The significant expression in CD may indicate the activation of mitophagy as an essential process to prevent the adverse effects of inflammation, during which reactive oxygen species (ROS) damage mitochondria, which exacerbates the process.

Based on *PINK1* gene expression, our study indicated that mitophagy processes were highly active in CD. Xu Y et al. [28] also showed the protective role of mitophagy in IBD progression, including CD. Furthermore, emerging evidence indicates that *PINK1* functions as a tumor suppressor in CRC by modulating mitochondrial quality control and cellular metabolism. Through the activation of *TP53* signaling, *PINK1* promotes mitophagy, leading to the clearance of damaged mitochondria, a reduction in glycolytic activity, and an enhancement of mitochondrial oxidative phosphorylation. This metabolic reprogramming counteracts the Warburg effect, inhibiting tumor progression [66].

Carcinogenesis-related oxidative stress and inflammatory processes in CRC also stimulate mitophagy, albeit more weakly and impaired expression of the gene encoding *PINK1* caused by mutation accelerates tumor growth in CRC [58,65]. *PINK1* seems to have a similar tumor suppressor role in pancreatic cancer [67]. Although mitophagy was low or even dormant at the initial stage of carcinogenesis in CRC, its levels increased in later stages in our study. The comparison of *BECN1* and *PINK1* expressions showed a slight negative correlation between the two proteins. It seems that the suggested cleansing and anti-tumor features of *BECN1* protein are fully utilized by the body at the initial stage of carcinogenesis processes.

However, the development of the disease and the breakdown of its protective function in CSII may increase inflammation, oxidative stress, and mitochondrial damage, which may result in increased protective mitophagy. It can be explained by the periodically increased expression of *PINK1* (CSII, CSIV) at crucial moments in the development of local advancement and dissemination. In our study, mitophagy and *PINK1* expression were increased in both inflamed and tumor cells. These results do not prove causal or mechanistic relationships, and that further validation using protein-level analyses and functional assays is necessary to clarify the potential role of these genes in the pathogenesis of Crohn’s disease and colorectal cancer. The increased expression levels in these tissues and a negative correlation with the *BECN1* protein (elevated in healthy tissues) suggest that mitophagy may be a process that mainly counteracts the ongoing disease, as opposed to macroautophagy, which can prevent neoplasia in inflammatory diseases.

This finding indicates a clear link between intestinal inflammation and the strength of *PINK1* gene expression. Also, it suggests an important and unfavorable role of mitophagy disruption in CRC, whose activity decreases in cancer. Therefore, a decrease is seen in the effectiveness of protection against the adverse effects of inflammation and adverse changes in the tumor microenvironment.

The *LAMP2* protein is an integral component of the membrane of lysosomes and plays a crucial role in CMA. It acts as a receptor that transports selected proteins into the lumen of the lysosome. The consequence of genetic or epigenetic silencing of *LAMP2* is related to impaired CMA and the potential accumulation of proteins that would be removed in healthy cells. It could include proteins that regulate the cell cycle or immune response. Therefore, abnormalities in this process have been linked to autoimmune diseases and cancer [68].

Electron microscopy studies of the intestines in IBD indicated no significant difference in the distribution of *LAMP2* in enterocytes from the duodenum to the colon. Furthermore, no differences were found in the ileum or large intestine between healthy and affected tissue samples [64]. *LAMP2* was present in CD in healthy intestinal mucosa, affected areas, CRC, and the precancerous stages of CRC [69].

Studies indicate that CMA processes are increased in some cancers. Additionally, overexpression of *LAMP2* in cancers (e.g., liver or breast carcinoma) is associated with a poor prognosis [68,70,71]. However, as in our study, reduced levels of *LAMP2* expression in colon cancer were observed in tumor tissues compared to healthy colon mucosa [72,73].

The importance of *LAMP2* in antigen presentation and regulation of the immune response suggests that *LAMP2* may modulate the course of IBD through changes in the efficiency of bacterial antigen removal and control of intestinal inflammation [71,74]. However, the data are inconclusive. At certain stages of IBD development or progression, *LAMP2* expression may be increased, especially in selected immune cell populations [75].

Our study found the lowest median expression of *LAMP2* protein in the CRC group. It was slightly lower than in CD and significantly lower than in controls. The results of our study did not show excessive activity of CMA processes in tumor tissues. It suggests that the CMA pathway may be inhibited in CRC, which could be due to tumor adaptation to chronic metabolic or oxidative stress, which allows temporal “tolerance” of some damaged proteins [76,77]. However, median *LAMP2* expression in CD tissues was lower than in normal tissues. However, it was not statistically significant. The highest mean with a high standard deviation was observed in patients with CD, which is due to the heterogeneity of the group in terms of disease activity (endoscopically diagnosed patients with a different medical history, surgical patients with a long course of the disease, different CDAI score levels). This indicates significant changes in the activity of this protein at different stages of CD, which may suggest that *LAMP2* could be a potential marker for assessing CD activity.

The mechanism of CMA is more complex as impairment of its activity may result from galectin deficiency in *LAMP2* and impaired autophagy. The structure of *LAMP2* in cancer cells is characterized by a marked difference in oligosaccharide structure compared to non-malignant cells [71]. Cells appear to be less efficient at housekeeping functions and antigen presentation, which may promote chronic inflammation [69].

*LAMP2* is one of the main components of the membrane of lysosomes and is one of the most glycosylated proteins of several cell types, including cancer cells. Tumor cells use CMA to degrade antiproliferative proteins, tumor suppressor proteins and proapoptotic proteins (e.g., *TP53*, *BCL-2*), thus increasing tumor growth [78,79,80,81,82,83].

A strong positive correlation between *PINK1* and *LAMP2* in healthy tissues, which was not found in CD or CRC, indicates a profound dysregulation of mechanisms of cell quality control under pathological conditions.

## 4. Conclusions

This study demonstrates altered expression of selected autophagy-related genes in colorectal cancer and Crohn’s disease tissues compared with non-diseased controls. Differential transcriptional patterns of *BECN1*, *PINK1*, and *LAMP2* were observed across colorectal cancer stages, suggesting that distinct autophagy pathways may be differentially regulated in inflammation-associated and sporadic colorectal tumorigenesis. Importantly, these findings are based on transcriptional analyses and therefore reflect associative rather than causal relationships. While changes in mRNA expression may indicate long-term alterations in autophagy regulation at the tissue level, functional consequences and protein-level effects were not assessed in the present study. Consequently, the observed differences should be interpreted with caution. Overall, our results contribute to a better understanding of autophagy-related transcriptional dysregulation in colorectal cancer within the context of chronic intestinal inflammation and should be regarded as hypothesis-generating. Further validation in larger cohorts, as well as protein-level and functional studies, is required to clarify the biological and potential clinical significance of these findings.

## Figures and Tables

**Table 1:** Clinical characteristics of patients with CD and CRC.

Parameter	CD Group *n* = 48				CRC Group *n* = 87				*p*
	Median	Range	Q1	Q3	Median	Range	Q1	Q3	
Age [y]	43.5	22–78	31.0	58.5	68.0	41–82	59.0	73.0	0.0001
Height [m]	1.70	1.54–1.94	1.63	1.77	1.66	1.54–1.88	1.56	1.76	n.s.
Body mass [kg]	62.0	35–107	56.5	72.5	76.0	45–105	63.0	87.0	0.0001
BMI (kg/m^2^)	21.85	13.5–28.43	19.42	24.20	26.45	18.17–36.73	24.45	29.50	0.0001
Ht [%]	36.1	25.1–47.4	30.4	40.5	37.9	27.2–47.8	36.2	40.1	n.s.
WBC [M]	7.12	3.60–25.1	4.84	9.67	6.56	2.90–15.76	5.09	8.37	n.s.

*n*—number of patients; BMI—Body mass index; Ht—hematocrit; WBC—white blood cell count, Q1/Q3—lower quartile/upper quartile; *p*—level of statistical significance; n.s.—not statistically significant.

**Table 2:** Distribution of patients by gender.

Gender	CD Group		CRC Group		*p*
	*n*	%	*n*	%	
Female	28	58.0	36	41.4	n.s.
Male	20	42.0	51	58.6	n.s.

*n*—number of patients; *p*—level of statistical significance; n.s.—not statistically significant.

**Table 3:** Analysis of the incidence of cancer stage in patients with CRC.

Cancer Stage	Number of Cases	%
Stage I	22	25.3
Stage II	20	23.0
Stage III	33	37.9
Stage IV	12	13.8
Total	87	100.0

**Table 4:** Sequences of the primers used for the RT-qPCR reaction.

Gene	Primer Sequences 5′ → 3′ (Forward)	Primer Sequences 5′ → 3′ (Reverse)
*BECN1*	CAGTATCAGAGAGAATACAGTG	TGGAAGGTTGCATTAAAGAC
*LAMP-2*	AACAAAGAGCAGACTGTTTC	CAGCTGTAGAATACTTTCCTTG
*PINK1*	TGTAAAACGACGGCCAGT	CAGGAAACAGCTATGACC

**Table 5:** Descriptive statistics of copy numbers of gene expression in samples from CD, CRC (regardless of tumor stage), and normal tissues.

Group/mRNA Molecules		Mean × 10^6^	Median	SD × 10^6^	Min	Max × 10^6^	IQR
CD	*BECN1*	0.01158	4062	0.01	0.02	0.093	16,610
	*PINK1*	1.40416	17,380	13.6	84.00	133.3	6388
	*LAMP2*	13,524.6	19,215	43,398.0	0.42	262,500	20,054
CRC	*BECN1*	11,074.5	6388	0.02	0.37	0.0933	11,529
	*PINK1*	1372.30	194	6793.4	0.00	47,380	7198
	*LAMP2*	1.43173	18,250	13.12	857.0	122.4	18,540
CT	*BECN1*	8.53022	10,560	57.6062	0.15	453.9	16,532
	*PINK1*	0.02261	13	0.17992	0.00	1.717	1292
	*LAMP2*	3.74573	34,135	20.3783	5442	122.4	37,385

SD—standard deviation; IQR—inter-quartile range (interquartile distribution, i.e., the difference between the lowest and the highest measurement).

**Table 6:** Comparison of gene expression in the CD, CRC, and control samples using the Kruskal–Wallis H test.

Gene/Group Patients		*n*	Mean Rank	Median	Q1	Q3	*H*	*p*	*η* ^2^	Post Hoc A
			mRNA Copy/μg RNA							
*BECN1*	CD	96	121.511	4062.000	280.500	16,890.000	9.421	0.01	0.030	CD vs. CT
	CRC	87	132.823	6388.000	1571.000	13,100.000				
	CT	87	156.433	10,560.000	3853.000	20,385.000				
*PINK 1*	CD	96	197.411	17,380.000	5929.000	21,952.000	89.920	<0.001	0.320	CD vs. CRC CD vs. CT
	CRC	87	119.332	194.000	0.571	7199.000				
	CT	87	91.701	13.201	0.073	1292.000				
*LAMP2*	CD	96	126.900	19,215.000	4596.500	24,650.000	7.540	0.02	0.020	CRC vs. CT
	CRC	87	122.500	18,250.000	10,770.000	29,310.000				
	CT	87	151.702	34,135.000	14,357.500	51,742.500				

*n*—number of patients; Q1/Q3—lower quartile/upper quartile; *p*—level of statistical significance; post hoc a—pairs for which post hoc differences were significant at *p* < 0.05. *H*—Kruskal–Wallis H test; *η*^2^—eta squared (effect size measure).

**Table 7:** Spearman’s correlations for relationships between gene expressions.

Group/Gene Expression		*LAMP2*	*PINK1*	*BECN1*
Whole sample	*LAMP2*	1		
	*PINK1*	0.16 **	1	
	*BECN1*	<0.01	−0.23 **	1
Crohn’s disease	*LAMP2*	1		
	*PINK1*	0.16	1	
	*BECN1*	0.01	0.11	1
Colorectal cancer	*LAMP2*	1		
	*PINK1*	0.28 **	1	
	*BECN1*	−0.07	−0.24 *	1
Control group	*LAMP2*	1		
	*PINK1*	0.51 **	1	
	*BECN1*	−0.04	−0.13	1

* *p* < 0.05; ** *p* < 0.01.

**Table 8:** Comparison of gene expression among samples with colorectal cancer of various stages.

Gene	CC Stage	*n*	Mean Rank	Median	Q1	Q3	*H*	*p*	*η* ^2^
			Copies of RNA Per µg of Total RNA (mRNA/µg RNA)						
*BECN1*	CSI	22	50.52	10,245	3121	18,260	4.21	0.240	<0.01
	CSII	20	43.00	6168	1925	9297			
	CSIII	32	43.22	6141	1233	15,448			
	CSIV	12	32.21	2629	268	10,828			
*PINK1*	CSI	22	36.14	1.4	0.17	1214	4.70	0.196	0.01
	CSII	20	49.60	2458	3.04	9385			
	CSIII	33	42.62	153	0.91	3015			
	CSIV	12	52.88	7203	239.24	3470			
*LAMP2*	CSI	22	38.68	14,735	8131	24,833	1.86	0.602	<0.01
	CSII	20	47.75	19,470	12,428	34,783			
	CSIII	33	46.33	19,830	9097	48,815			
	CSIV	12	41.08	17,775	14,363	21,498			

*n*—number of patients; Q1/Q3—lower quartile/upper quartile; *p*—level of statistical significance; *H*—Kruskal–Wallis H test; *η*^2^—eta squared (effect size measure).

## Data Availability

The original contributions presented in this study are included in the article. Further inquiries can be directed to the corresponding authors.

## Abbreviations

The following abbreviations are used in this manuscript:

*BECN1*	the human gene encoding the Beclin-1 protein
CD	Crohn’s disease
CMA	chaperone-mediated autophagy
CRC	colorectal cancer
CSI, CSII, CSIII and CSIV	different stages of colon cancer
IBD	inflammatory bowel disease
*LAMP2*	Lysosome-associated membrane protein 2
mTOR	major cell proliferation pathways
*PINK1*	*PTEN-induced kinase 1*
UC	ulcerative colitis
